# Design of self-assembled glycopolymeric zwitterionic micelles as removable protein stabilizing agents†

**DOI:** 10.1039/d3na00002h

**Published:** 2023-02-21

**Authors:** Robin Rajan, Kazuaki Matsumura

**Affiliations:** a School of Materials Science, Japan Advanced Institute of Science and Technology 1-1 Asahidai Nomi Ishikawa 923-1292 Japan robin@jaist.ac.jp mkazuaki@jaist.ac.jp

## Abstract

Developing stabilizers that protect proteins from denaturation under stress, and are easy to remove from solutions, is a challenge in protein therapeutics. In this study, micelles made of trehalose, a zwitterionic polymer (poly-sulfobetaine; poly-SPB), and polycaprolactone (PCL) were synthesized by a one-pot reversible addition–fragmentation chain-transfer (RAFT) polymerization reaction. The micelles protect lactate dehydrogenase (LDH) and human insulin from denaturation due to stresses like thermal incubation and freezing, and help them retain higher-order structures. Importantly, the protected proteins are readily isolated from the micelles by ultracentrifugation, with over 90% recovery, and almost all enzymatic activity is retained. This suggests the great potential of poly-SPB-based micelles for use in applications requiring protection and removal as required. The micelles may also be used to effectively stabilize protein-based vaccines and drugs.

## Abstract 2

Polysulfobetaine, trehalose, and polycaprolactone were combined to create micelles that stabilize proteins and can be easily removed by ultracentrifugation. The three components work synergistically to enhance protein protection within the micelles.

## Graphical abstract

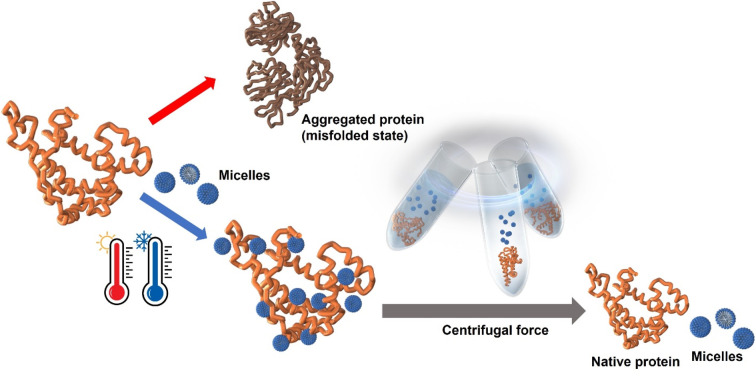

## Introduction

The development of removable protein stabilizers is the ultimate goal in the preclinical phase of protein biopharmaceutics. The field of protein-based therapeutics is hampered by the frequent denaturation or aggregation of proteins in all stages of protein product development and manufacturing, including protein expression, formulation, purification, product filling, lyophilization, transport, and storage.^1^ Protein denaturation is usually triggered by environmental stresses, such as high temperatures, freezing, and pH changes.^2–4^ Denaturation is a major concern in protein-based biopharmaceutics.^5–7^ Additionally, several neurodegenerative diseases, such as Alzheimer's and Parkinson's, have been linked to protein aggregation.^8,9^ Hence, it is imperative to keep proteins in their native state.

A number of compounds and techniques have been developed to combat protein denaturation and aggregation.^5,10–14^ Recently, many polymers, including polyethylene glycol,^15^ glycopolymers,^16^ and conjugated polymers,^17,18^ have been employed for suppressing protein aggregation with very high efficiency. However, these compounds are difficult to remove from protein solutions, which impedes their application in protein-based drugs. To employ proteins for biotherapeutic applications or to administer to a patient, it is preferable to use them in their native form without any additives (such as the polymers that are used to stabilize them); hence, it is necessary to develop a method to remove the stabilizing agent from the protein. Most previous studies have focused on the development of novel protein aggregation inhibitors, but little work has been dedicated toward the removal of stabilizing agents from proteins. Hence, developing a protein stabilizer that preserves the protein's structure and activity and can later be separated from the protein sample is expected to be of great significance in the field of protein therapeutics.

In our previous studies, we showed that poly-sulfobetaine (poly-SPB), a zwitterionic polymer, exhibits good efficiency in protecting proteins from thermal denaturation.^19–24^ A number of poly-SPB derivatives have been prepared by adding hydrophobic monomers, transforming into nanogels, or preparing graft polymers to achieve higher activity. Although previously known agents and zwitterionic polymers have remarkable abilities to protect proteins, a major drawback of this process is the inability to separate proteins from the stabilizing agents. Proteins and reagents are usually dissolved in an aqueous buffer to form a homogeneous solution, which prevents the removal of the stabilizing agents from the protein after they are mixed. The ability to successfully isolate proteins from the stabilizing agent will make these materials attractive for use in the development of protein-based drugs, as well as for safe transportation and storage of proteins. This is especially urgent given the pressing need to develop agents that can stabilize protein-based vaccines and drugs, as well as other similar products. Additionally, these systems can be employed during various protein or antibody processing steps to prevent denaturation and can be safely removed when the process is complete. Another critical issue is the design of an easy removal process that does not cause protein denaturation.

**Fig. 1:**
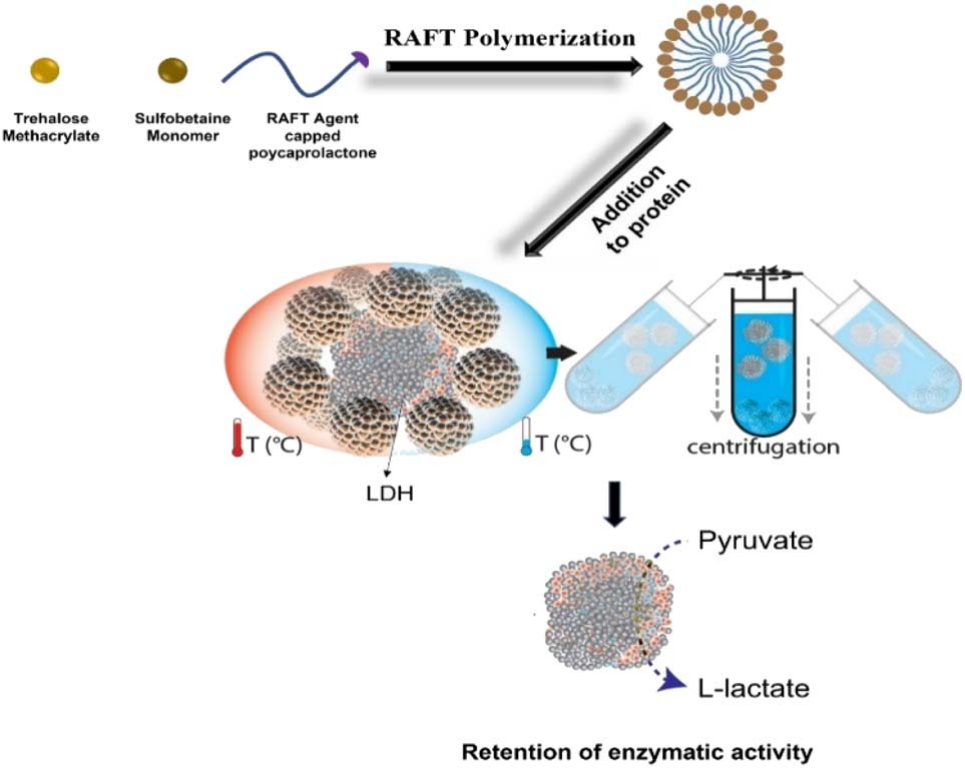
Schematic illustration representing the preparation of the self-assembled micelles and subsequent protection of proteins (from thermal and cold stress) and removal using ultracentrifugation.

As a proof of concept, in this study, we developed self-assembled micelles that protect proteins from different types of stress and can later be separated by a simple ultracentrifugation step (Fig. 1).^25,26^ Micelles were prepared using poly-SPB, and trehalose was added to enhance the stabilizing ability^27–29^ and improve the dispersibility of micelles in the aqueous buffer. A recent study revealed that trehalose stabilizes insulin from denaturation by inhibiting the deamination of amino acid residues as well as suppressing its fibrillation, then aggregating inactive and immunogenic amyloids without complexing insulin into its hexameric state, thus possibly delaying the commencement of insulin activity.^30^ Additionally, polycaprolactone (PCL) was introduced to induce self-assembly. Protein stability studies were carried out using lactate dehydrogenase (LDH) and recombinant human insulin under several types of stress. Pure proteins in their native state with complete activity were then isolated from the protein–micelle mixture.

## Experimental section

### Materials and methods

SPB was donated by Osaka Organic Chemical Industry, Ltd (Osaka, Japan) and used without further purification. d-(+)-Trehalose dihydrate was purchased from Nacalai Tesque (Kyoto, Japan). Tin(ii) 2-ethylhexanoate and ε-caprolactone were purchased from Tokyo Chemical Industry Co., Ltd (Tokyo, Japan). 4-Cyano-4-[(dodecylsulfanylthiocarbonyl)-sulfanyl]pentanol (RAFT agent), V-501 initiator (4,4-azobis(4-cyanovaleric acid)), lactate dehydrogenase (from rabbit muscle), anhydrous triethylamine, sodium pyruvate, β-nicotinamide adenine dinucleotide (reduced disodium salt hydrate), and methacrylic anhydride were purchased from Sigma-Aldrich Corp. (St. Louis, MO, USA). Insulin (human) was purchased from Roche Diagnostics GmbH (Mannheim, Germany).

### Synthesis of trehalose methacrylate (TrMA)

Trehalose dihydrate was first dried at 105 °C under vacuum for 2 days. Dried trehalose (1 equiv.) was then mixed with anhydrous dimethylformamide, followed by the addition of anhydrous triethylamine (2 equiv.). Methacrylic anhydride (1 equiv.) was added dropwise to the reaction mixture, and the reaction mixture was purged with nitrogen gas for 30 min, followed by stirring at room temperature for 24 h in a nitrogen atmosphere.^31^ The reaction mixture was precipitated in cold diethyl ether, and the product was washed repeatedly with hexane and diethyl ether. The purified product was obtained by drying under vacuum.

### RAFT-agent-initiated ring opening polymerization of caprolactone (PCL-CTA)

The RAFT agent (1 equiv.) was dissolved in anhydrous DMF. To this, ε-caprolactone (30 equiv.) and tin(ii) 2-ethylhexanoate (0.1 wt%) were added, and the reaction was allowed to proceed at 110 °C for 16 h under a nitrogen atmosphere. The reaction mixture was precipitated in a 1 : 1 (v/v) mixture of methanol and diethyl ether, and this process was repeated three times. The product was then obtained by drying under vacuum.

### Synthesis of PCL-*b*-(PSPB-*r*-PTrMA)

PCL-CTA and V-501 were dissolved in DMF in a round-bottom flask. In separate flasks, TrMA and SPB monomers were dissolved in DMSO and water, respectively. This was followed by the dropwise addition of TrMA and SPB solutions to PCL-CTA. The reaction mixture was then purged with N_2_ gas for 1 h, followed by stirring at 70 °C for 24 h. As an example, for the synthesis of M1; 0.0.2 mmol of PCL-CTA (0.167 g), 2 mmol SPB monomer (0.5565 g), 2 mmol of TrMA (0.821 g), 0.8 mg AIBN, and 60 mL solvent were used. After the reaction, the solvents were removed under vacuum, and the solid residue was washed with DMF to remove any unreacted PCL-CTA. The product was then further purified by dialysis (MWCO of 100 kDa, Spectra/Por® 3 Dialysis Membrane, Spectrum Labs, Inc., Rancho Dominguez, CA, USA) against deionized water for 3 days. A 100 kDa dialysis membrane was used to ensure that all unreacted monomers or homopolymers of the SPB monomer and TrMA were removed. The final product was obtained by lyophilization.

### Dynamic light scattering and zeta-potential

The micelles were prepared at a concentration of 4 mg mL^−1^ in deionized water and the particle charge and size were measured using a Zetasizer 300 system (Malvern Instruments, Worcestershire, UK) in a DTS1070 folded capillary cell. The scattering angle was 173°, and the temperature was set to 25 °C.

### Critical micelle concentration

The critical micelle concentrations of self-assembled micelles were determined using pyrene as a hydrophobic fluorescent probe. Fifteen microliters of pyrene solution (0.05 mM in acetone) were added into several Eppendorf tubes, followed by drying under a gentle stream of nitrogen gas. Micelle suspensions (0.5 mL) of various concentrations were added to each tube and allowed to stand at 25 °C in the dark for 18 h. The sample (150 μL) was then added to a glass-bottom 96-well plate in triplicate. The fluorescence of the samples was measured using a microplate reader (Tecan Infinite 200 PRO M Nano^+^) at an excitation wavelength of 339 nm, and emission spectra were collected at 373 and 394 nm.^32^

### Transmission electron microscopy (TEM)

Twenty microliters of micelles (0.25 mg mL^−1^) were placed on a copper grid (NS-C15 Cu150P; Stem, Tokyo, Japan). The grid was negatively stained with 1% phosphotungstic acid (Sigma-Aldrich, Steinheim, Germany) for 45 s and then air-dried. TEM images were collected using a Hitachi H-7650 system (Hitachi, Tokyo, Japan) with an accelerating voltage of 100 kV.

### Isothermal titration calorimetry (ITC)

Experiments were performed with a MicroCal PEAQ-ITC (Malvern) using MicroCal PEAQ-ITC Control Software. The sample cell was filled with 200 μL of protein, and the titrant syringe was filled with 40 μL of the different polymers in the same buffer as the protein. The reference cell was filled with deionized water. For each experiment, 40 μL of titrant was loaded into the titration syringe and for the titration, initially 0.2 μL was injected to the cell followed by addition of 2 μL 19 times at a time interval of 150 s. The following settings were used: stir speed 750 rpm, temperature 25 °C, initial delay 60 seconds.

### LDH aggregation assay

UV-visible (UV-Vis) spectra were obtained to investigate the aggregation of LDH with and without different additives.^33^ LDH solution (0.2 mg mL^−1^) in phosphate-buffered saline (PBS) (pH 7.4) was mixed with different concentrations of micelles and transferred to a 1 mL quartz cuvette, and the absorbance was recorded at 350 nm on a UV-Vis spectrophotometer (UV-1800, Shimadzu Corp., Kyoto, Japan) at 37 °C over a period of 30 min with constant stirring.

### Circular dichroism (CD) spectroscopy

The CD spectra of LDH in PBS were obtained to record the changes in the secondary structure of the protein before and after incubation with various additives (JASCO-820 spectropolarimeter). The final concentration of LDH and path length were 0.1 mg mL^−1^ in PBS (pH 7.4) and 1 cm, respectively. The CD spectrometer was purged with nitrogen gas for 30 min before starting the experiments and attached to a chiller maintained at 15 °C. Each spectrum was baseline-corrected and collected as an average of three scans.

### LDH activity assay (incubation)

Micelles and LDH (20.7 mU mL^−1^ in PBS) were mixed and incubated at 37 °C for 1 h in a BioShaker (BR-40LFA, Taitec Corporation, Japan). Each incubated sample (5 μL) was placed in a 96-well plate. A stock solution of sodium pyruvate and NADH was prepared in PBS at concentrations of 10 and 63 mM, respectively.^34^ A master mix was prepared immediately before use by adding 200 μL of NADH and 500 μL of sodium pyruvate to chilled PBS (49.3 mL). Then, 195 mL of the master mix was added to each well and mixed well. Absorbance at 340 nm was recorded at different time intervals using a Tecan Infinite 200 PRO M Nano^+^ microplate reader. The LDH activity was evaluated by observing the rate of decrease in absorbance with respect to the fresh (non-incubated) LDH solution over a period of 10 min.

### LDH activity assay (freeze–thaw)

Micelles and LDH (20.7 mU mL^−1^ in PBS) were mixed and frozen by plunging in liquid nitrogen for 3 min, followed by thawing at 25 °C for 5 min. This process was repeated 15 times to complete 15 freeze–thaw cycles.^35^ The LDH activity was evaluated using a method similar to that described above.

### LDH removal

After the incubation of LDH with or without micelles, they were isolated by ultracentrifugation at 1.32 × 10^4^ rpm (5 °C) for 90 min, leading to precipitation of the micelles. LDH was then collected from the supernatant.

### Bradford assay

Micelles (2 mg mL^−1^) were mixed with LDH to achieve a final LDH concentration of 75 μg mL^−1^, followed by incubation at 37 °C for 1 h. The incubated samples were then centrifuged at 1.32 × 10^4^ rpm (5 °C) for 90 min, and the clear supernatant was collected. Ten microliters of each supernatant were loaded onto a 96-well pate followed by the addition of 200 μL of Bradford reagent (Takara Bio Inc., Japan) to each well. The samples were incubated for 10 min in the dark, and the absorbance at 595 nm was recorded using a microplate reader.

### Mass spectroscopy

Mass spectra were obtained in the positive linear mode using a MALDI-TOF MS (ultrafleXtreme, Bruker Daltonics, Bremen, Germany) with 2-[(2*E*)-3-(4-*tert*-butylphenyl)-2-methylprop-2-enylidene]malononitrile (DCTB) as the matrix and sodium trifluoroacetate (TFANa) as the cationic reagent.

### Thioflavin T (ThT) assay

ThT stock solution was prepared by adding 4 mg of ThT to 5 mL of PBS and filtering through a 0.22 μm filter. The stock solution was diluted by adding 1 mL of the stock solution to 49 mL of PBS to obtain the working solution. Human insulin solution in PBS was mixed with various micelles and incubated at 45 °C for 72 h in a BioShaker. Then, 0.1 mL of this solution was mixed with 2 mL of the working solution of ThT, and fluorescence was observed at an excitation wavelength of 450 nm and emission wavelength of 484 nm (JASCO FP6500).^36^ The increase in intensity corresponds to amyloid formation due to the binding of ThT to amyloid filaments.

### Cytotoxicity studies

L929 cells were cultured in Dulbecco's modified Eagle's medium supplemented with 10% heat-inactivated fetal bovine serum in a humidified atmosphere of 5% CO_2_ at 37 °C. Cytotoxicity assay was performed according to the MTT method. Briefly, 0.1 mL of 1 × 10^4^ cells per mL was seeded in a 96-well plate and incubated for 72 h at 37 °C, followed by the addition of 0.1 mL of micelles at different concentrations, dissolved in the culture medium. The micelle-containing cells were then re-incubated for 24 h at 37 °C, and 100 μL of 3-(4,5-dimethylthiazole-2-yl)-2,5-diphenyltetrazolium bromide (MTT) solution (300 μg mL^−1^) was added to each well, and the cells were incubated again for 3 h. After carefully discarding the media, 0.1 mL of DMSO was added to each well to dissolve the purple formazan crystals that formed as a result of the reduction of MTT. The absorbance values at 540 nm were recorded using a Tecan Infinite 200 PRO M Nano^+^ microplate reader.

### Statistical analysis

All data are expressed as the mean ± standard deviation (SD). All experiments were conducted in triplicate. To compare the data, ordinary one-way analysis of variance with Tukey's multiple comparison test was used. Differences were considered statistically significant at *p* < 0.05.

## Results and discussion

### Synthesis and characterization

**Scheme 1:**
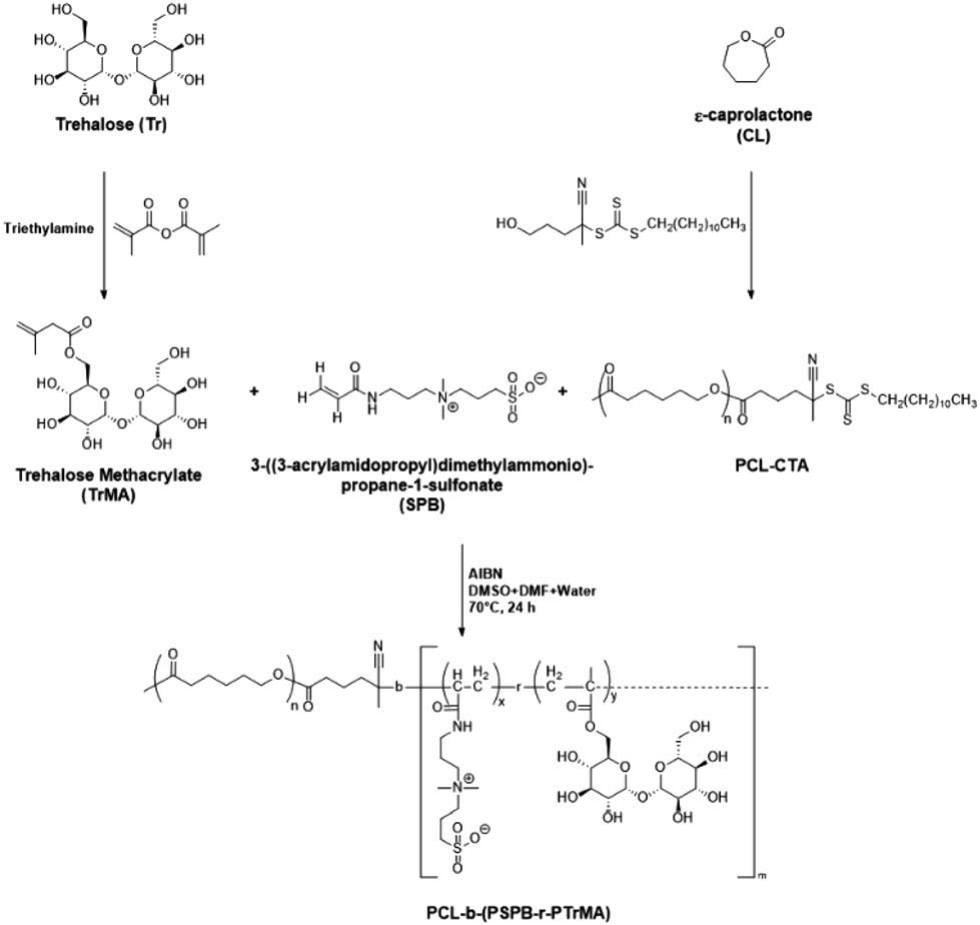
Synthesis of glycopolymeric zwitterionic micelles by one-pot RAFT polymerization.

In the first step of the micelle preparation process, trehalose was rendered polymerizable by incorporating a methacrylate group *via* the controlled addition of methacrylic anhydride in the presence of triethylamine (Scheme S1†). Substitution of the methacrylate group was determined by ^1^H nuclear magnetic resonance (NMR) and Fourier-transform infrared (FTIR) spectroscopy. The presence of vinyl peaks in the ^1^H NMR spectra and a C=O peak at 1713 cm^−1^ in the FTIR spectra confirmed the formation of trehalose methacrylate (TrMA) (Fig. S1–S4†). Meanwhile, ring-opening polymerization of caprolactone was carried out using a hydroxyl-terminated reversible addition–fragmentation chain-transfer (RAFT) agent in the presence of stannous(ii) octoate to yield a PCL-based macro-chain transfer agent (PCL-CTA) (Scheme S2†). The presence of the C=S_str_ peak at 1069 cm^−1^ in the FTIR spectra confirmed the formation of RAFT-agent-terminated PCL, which was subsequently used for the preparation of micelles (Fig. S5†). ^1^H and ^13^C-NMR spectroscopic studies further confirmed the formation of the RAFT-agent-terminated PCL (Fig. S6 and S7†). The methyl group peak corresponding to the terminal-end RAFT agent was used to determine the degree of polymerization of caprolactone and was found to be 69. In the final step, a one-pot RAFT polymerization was carried out using TrMA, SPB monomer, and PCL-CTA to yield a tripolymer composed of all three components (Scheme 1). Owing to the presence of both the hydrophobic chain (PCL) and the hydrophilic branches (poly-trehalose and poly-SPB), the resultant polymer self-assembled when dispersed in water to form micelles. FTIR spectroscopy of the prepared micelles suggested that all three components were successfully incorporated into the micelles (Fig. S8†).

**Fig. 2:**
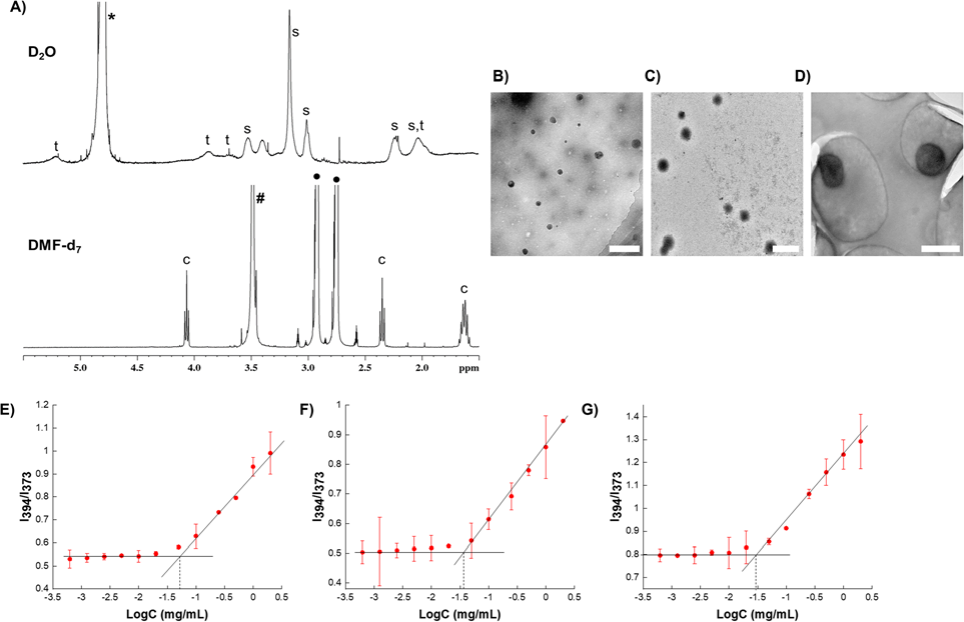
Characterization of micelles. (A) ^1^H NMR of micelle in D_2_O and DMF-d_7_; s, t and c indicate the peaks of the repeating units of SPB, trehalose and caprolactone, respectively. TEM image of (B) M1, (C) M2, and (D) M3 (scale bars represent 500 nm). Plots of *I*_394_/*I*_373_ in excitation spectra as a function of log *C* for (E) M1, (F) M2, and (G) M3. Errors bars indicate standard deviation of the mean.

To demonstrate the formation of micelles, ^1^H NMR spectra of the micelles were collected in both D_2_O (aqueous solvent) and DMF-d_7_ (organic solvent). Contrasting spectra were obtained (Fig. 2A). In D_2_O, sharp peaks corresponding to SPB and trehalose (hydrophilic repeating units) were observed, indicating their high diffusibility in aqueous media. Caprolactone (hydrophobic) peaks were too broad to be observed, which could be due to its aggregation in aqueous media.^37,38^ In contrast, in DMF-d_7_, sharp signals of caprolactone appeared and the peaks of SPB and trehalose disappeared or reduced in intensity. This behavior unambiguously demonstrates the presence of both hydrophobic and hydrophilic components in the polymer, where the hydrophobic part is masked in aqueous media, thus confirming the formation of micelles.

**Fig. 3:**
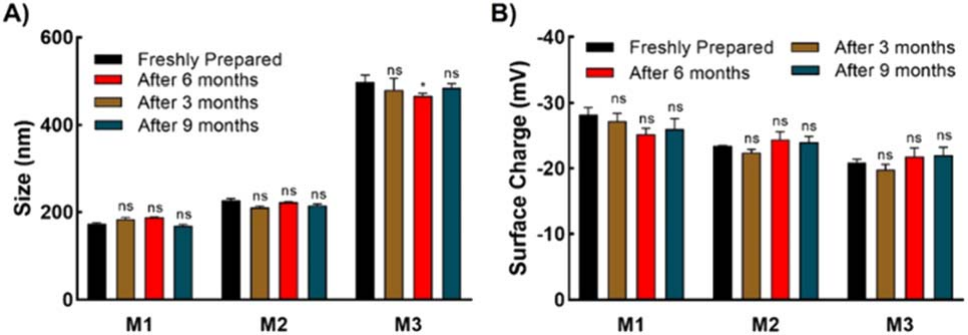
Stability of aqueous suspension of micelles. (A) Size of micelles obtained at different times using dynamic light scattering, and (B) zeta-potential of micelles obtained at different times. Errors bars indicate standard deviation of the mean. The significances, which were calculated against the freshly prepared suspensions for each micelle, are marked as *: *p* < 0.05, ns: not statistically different.

Three types of micelles (denoted as M1, M2, and M3) were prepared with different ratios of trehalose, SPB, and PCL-CTA. Their compositions and characteristics are summarized in Table 1. Transmission electron microscopy (TEM) revealed the formation of spherical micelles with sizes ranging from to 160–365 nm (Fig. 2B–D). Dynamic light scattering (DLS) also demonstrated the formation of monodisperse micelles with hydrodynamic diameters of 170–450 nm (Fig. S9†). The micelles showed a negative charge ranging from approximately −20 to −30 mV (Fig. S10†), and the nanosized micelles were stable in an aqueous suspension at 25 °C with no appreciable change in size or surface charge for up to nine months (Fig. 3). The critical micelle concentration, which is the minimum concentration at which micelle formation takes place, was evaluated using the pyrene fluorescence method.^32^ The three types of micelle had critical micelle concentrations of 28–48 μg mL^−1^ (Fig. 2E–G, Table 1), suggesting the formation of self-assembled aggregates.

**Table 1:** Characteristics of micelles M1–M3 prepared *via* reversible addition–fragmentation chain-transfer (RAFT) polymerization

	[R] : [C_1_] : [C_2_]^a^	Size^b^		Surface charge^c^ (mV)	Critical micelle concentration^d^ (μg mL^−1^)
		DLS (nm)	TEM (nm)		
M1	[1] : [100] : [100]	173.9 ± 1.997	158.2	−28.2 ± 1.10	47.9
M2	[1] : [200] : [100]	227.3 ± 3.821	198.2	−23.4 ± 0.10	36.3
M3	[1] : [100] : [500]	497.5 ± 15.68	364.3	−20.9 ± 0.513	28.2

a Concentrations of R: polycaprolactone (PCL)-RAFT agent, C_1_: SPB monomer, and C_2_: trehalose methacrylate (TrMA).

b Micelle size determined by dynamic light scattering (DLS) and transmission electron microscopy (TEM).

c Determined by zeta potential measurement.

d Determined by pyrene fluorescence method.

### Protein aggregation inhibition

**Fig. 4:**
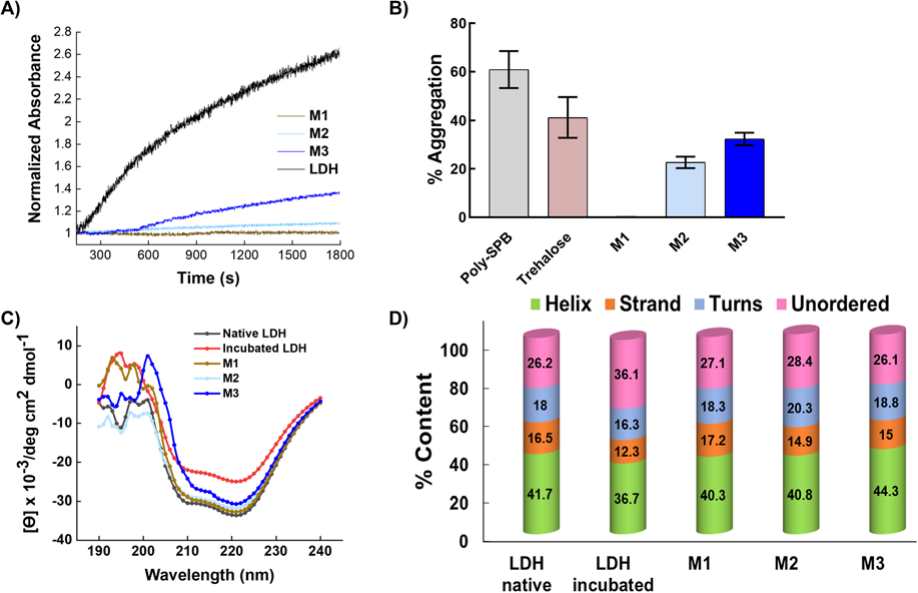
LDH aggregation after incubation. (A) Aggregation of LDH in the presence of micelles (2 mg mL^−1^), evaluated by measuring the turbidity of the solution at 350 nm as a function of time at 37 °C, (B) LDH aggregation when incubated in presence of various additives at 37 °C for 1 h, (C) representative far-UV CD spectra of insulin in the presence of various micelles (2 mg mL^−1^) after incubation, and (D) percentages of secondary structure contents.

To investigate the protein protection property, LDH was used as a model protein. LDH undergoes thermal- and freezing-induced aggregation. When LDH was incubated at 37 °C for 30 min, an increase in the light scattering of the LDH solution was detected, indicating that native LDH transformed into large, insoluble aggregates.^33,39^ However, when LDH was mixed with the micelles prior to incubation, the rate of increase in absorbance was markedly lower than when no additives were used, clearly showing that the micelles suppressed the aggregation of LDH (Fig. 4A and B). In the solution with added M1, negligible increases in light scattering were observed during the incubation period, indicating it had the highest efficiency (around 0.02% aggregation at 2 mg mL^−1^). M2 and M3 had slightly lower efficiency, which may be because M1 is smaller than the other micelles, or because the amounts of poly-SPB and trehalose were more favorable for suppressing protein aggregation. Importantly, all micelles showed significantly higher stabilizing behavior than poly-SPB and trehalose alone. This high efficiency can be explained by various factors: the synergistic effects of poly-SPB and trehalose (both well-established protein protection agents), the presence of hydrophobicity (which has been reported to significantly augment the protection ability^21^), and the small size of the particles (which has been attributed to greater shielding from aggregation-inducing collisions in proteins).

The effect of the micelles on the higher-order structure of proteins was investigated using circular dichroism (CD) spectroscopy. Upon incubation (with stirring) at 37 °C for 30 min, the LDH solution displayed a significant decrease in the intensity of the CD bands around 210 and 222 nm, corresponding to β-sheets and α-helices, respectively.^15^ However, the addition of micelles resulted in retention of the CD bands (Fig. 4C), suggesting that the micelles help to retain the secondary structure of LDH, even when subject to thermal stress. The results correspond well with the thermal aggregation assay using UV-Vis spectroscopy, in which micelles protected LDH from aggregation and M1 showed the highest efficiency. To further confirm the structural changes, quantitative analysis of the secondary structural elements was performed using the SELCON3 program.^40,41^Fig. 4D clearly shows that when LDH was incubated without any additives, its secondary structure—especially the amount of unordered conformation—changed significantly. However, the addition of micelles to LDH prior to heating yielded almost identical secondary structure elements (Fig. 4D) to those of native LDH, indicating that micelles preserve the higher-order structures of LDH, which, in turn, suppresses the thermal aggregation of LDH.

#### LDH activity

**Fig. 5:**
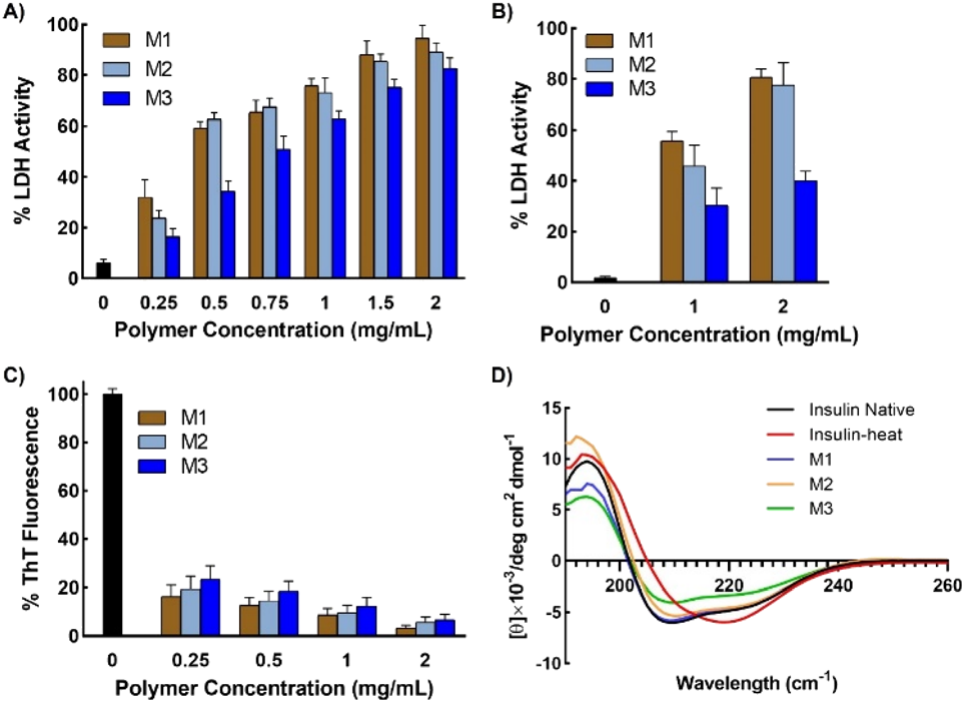
Protein activity after being subjected to stress. (A) Enzymatic activity of LDH after incubation in presence of micelles at 37 °C for 1 h, and (B) enzymatic activity after subjecting LDH in presence of micelles to 15 freeze–thaw cycles, (C) thioflavin T (ThT) fluorescence of human insulin when incubated at 45 °C for 72 h in the presence of micelles, and (D) representative far-UV CD spectra of human insulin after incubation at 45 °C for 72 h in the presence of micelles at 2 mg mL^−1^ concentration. Data are expressed as the mean ± SD of 3 independent experiments.

The LDH activity was evaluated by monitoring its ability to catalyze the conversion of β-nicotinamide adenine dinucleotide (β-NADH) to β-NAD in the presence of pyruvate, which manifests as a sudden decrease in absorbance at 340 nm.^42^ Incubated LDH solution (with or without micelles) was added to a mixture of NADH and pyruvate, and the change in light scattering was monitored over time. The results clearly showed that the addition of micelles to LDH prior to incubation resulted in the protein retaining almost all of its native enzymatic activity (Fig. 5A). The addition of a small amount (2 mg mL^−1^) of micelles resulted in complete retention of the enzymatic activity of LDH. Interestingly, the addition of even 0.5 mg mL^−1^ micelles enabled the retention of almost 60% enzymatic activity. A similar trend was observed for all three micelles, as observed in the aggregation studies. The ability of the micelles to protect LDH was significantly higher than that of a poly-SPB homopolymer and copolymers of poly-SPB and trehalose or caprolactone (Fig. S11†). This indicates that there was positive synergy between poly-SPB, PTrMA, and PCL, resulting in higher activity than that with the individual components. The activity of M1 was more than 10 times higher than that with previously reported inhibitors, poly-SPB^19^ and l-arginine^43^ (Fig. S12†), indicating the remarkable efficiency of the micelles to protect proteins from denaturation.

Further, to check the efficiency of micelles in stabilizing proteins under different forms of stress, LDH was incubated in the presence of micelles at 37 °C for 1 h and then subjected to 15 freeze–thaw cycles. Freeze–thaw cycling damages proteins, cells, and tissues. However, the results showed that remarkably high activity was retained in the presence of micelles, even after exposure to such harsh conditions (Fig. 5B). LDH retained more than 80% activity when freeze–thawed in the presence of micelles, thus demonstrating the potential of the micelles for the long-term storage of therapeutic proteins or other drugs under freezing conditions.

#### Human insulin protection

To check whether the micelles could protect other proteins, recombinant human insulin was heated in the presence of micelles. Thioflavin T (ThT) assay results clearly demonstrated that the micelles effectively suppress the aggregation of insulin, with less than 5% fibrillation observed at a micelle concentration of 2 mg mL^−1^ (Fig. 5C). The micelles were also able to help in the retention of the secondary structure of insulin; insulin that was mixed with micelles prior to heating yielded similar CD results to those of the native protein (Fig. 5D and S13†). This suggests the micelles provide good protection from aggregation and structural change. Thus, compared to our previous report, these micelles show markedly higher protection of both insulin (almost ten-fold higher activity) and LDH.^21,35^

**Fig. 6:**
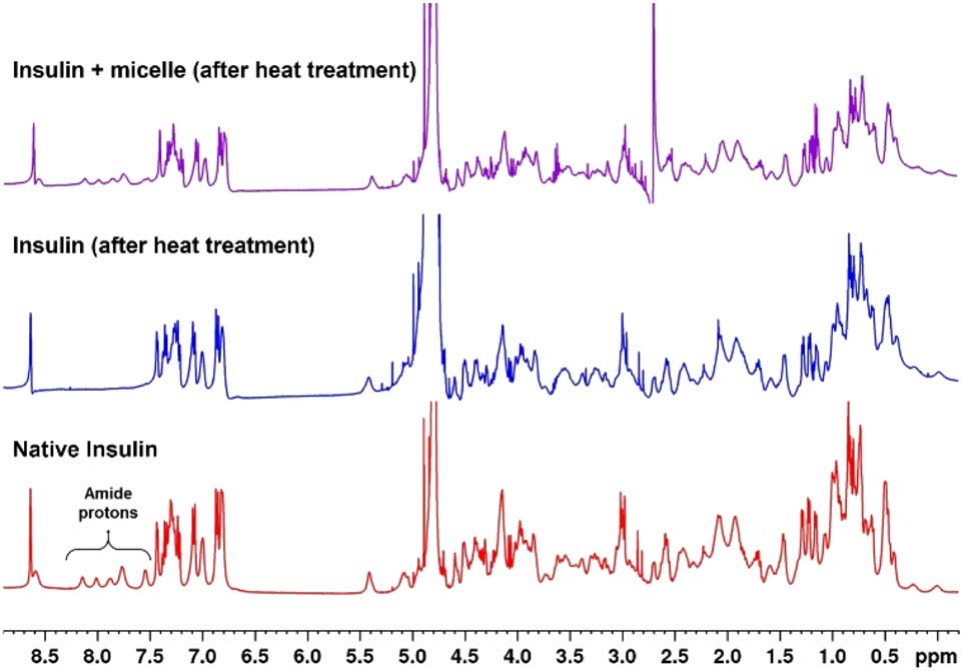
Comparison of ^1^H NMR of insulin with micelles in D_2_O before and after incubation at 45 °C for 72 h.

^1^H NMR spectra of native and heat-treated insulin with and without micelles were obtained to investigate the structural changes taking place in insulin on denaturation. Fig. 6 shows that the peaks between 7.5 and 8.2 ppm, which correspond to the amide protons of proteins, disappeared when the native insulin sample was subjected to heat treatment. This corresponds well with previous reports that protein denaturation triggers fast exchange of amide protons.^44–46^ In contrast, when micelles were added prior to heating, the amide peaks of the insulin sample were retained upon heating. This clearly demonstrates the role of micelles in suppressing protein denaturation.

### Separation of micelles

**Fig. 7:**
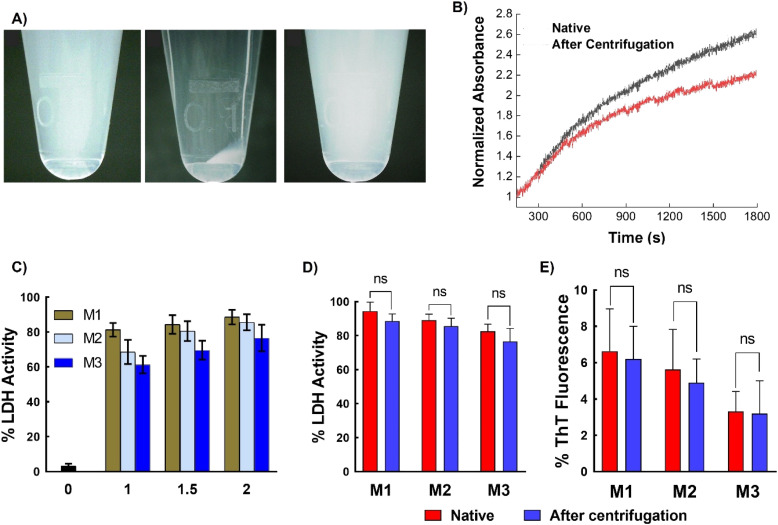
Removal of protein from micelles. (A) Photographs of micelles, left panel shows the micelle suspension in water, middle panel shows the micelles after ultracentrifugation, and the right panel shows the resuspended micelles after centrifugation, (B) aggregation of native LDH and LDH obtained after centrifugation, evaluated by measuring the turbidity of the solution at 350 nm as a function of time at 37 °C, (C) enzymatic activity of LDH after incubation with different micelles at different concentrations, (D) comparison of enzymatic activity of native LDH and LDH obtained after centrifugation (in the presence of micelles at 2 mg mL^−1^ concentration), and (E) comparison of ThT fluorescence of native human insulin and the protein obtained after centrifugation (in the presence of micelles at 2 mg mL^−1^ concentration). Data are expressed as the mean ± SD of 3 independent experiments. The significances, calculated against the freshly prepared suspensions for each micelle are marked as *: *p* < 0.05, ns: not statistically different.

Next, to assess the removability of the micelles from proteins, ultracentrifugation of LDH incubated with micelles was performed. The micellar structure allows for precipitation, which is not possible with simple polymers (non-micellar). The LDH solution mixed with different micelles was subjected to centrifugation at 1.32 × 10^4^ rpm, which resulted in the precipitation of micelles, while the native LDH remained in the supernatant. Careful removal of the supernatant and addition of water to the precipitate resulted in redispersion of the micelles (Fig. 7A). To confirm the absence of micelles in the supernatant, DLS was performed. Only an LDH peak was present, with no peaks corresponding to the micelles (Fig. S14†). This result unambiguously confirms the removal of micelles upon centrifugation. Complete removal of the micelles was further confirmed using matrix-assisted laser desorption/ionization time-of-flight mass spectrometry (MALDI-TOF MS), which showed that the recovered supernatant did not contain any traces of polymer (Fig. S15†). These results clearly show that ultracentrifugation successfully separates the micelles and proteins. The amount of LDH recovered was estimated using the Bradford assay, which showed that more than 90% of the protein was recovered (Fig. S16 and Table S1†). Thus, the micelles can be completely removed from the protein solution with good protein recovery.

Another important aspect is whether the recovered LDH retains its activity after heat treatment and subsequent centrifugation. To investigate this, the LDH recovered in the supernatant was incubated at 37 °C and the change in absorbance was monitored by UV-Vis spectroscopy. The recovered LDH sample showed similar behavior to native LDH (Fig. 7B), indicating that the collected sample retains its native properties. To further confirm this, the LDH activity of the recovered samples was examined. The protein displayed almost 90% of its native activity after heat treatment (37 °C for 1 h) and ultracentrifugation for 90 min (Fig. 7C). Similar behavior was observed with insulin—ultracentrifugation completely separated the micelles from the protein, with the zeta potential of the supernatant being almost the same (∼35 mV) as that of the native insulin solution, while the micelles alone showed negative zeta potential (Fig. S17†). Comparing the protein activity of the freshly prepared protein solution (native protein) with that of the recovered protein (after a heating–ultracentrifugation cycle in presence of micelles) revealed that the recovered protein retained similar activity to that of the native protein; that is, no significant changes in activity were observed (Fig. 7D and E).

These results can be explained by the weak interaction between the SPB-based micellar system and proteins. It has been well-established that zwitterionic polymers show weak and reversible interaction with proteins.^47^ This was corroborated by the ITC study to investigate the interaction between protein with poly-SPB. Fig. S18† shows that the exothermic peaks do not demonstrate any change in intensity upon injection of polymer to protein, thus indicating a very weak interaction exists between them. It should be noted that ITC data with micellar structure could not be obtained due to the limitation of the technique, albeit these results clearly demonstrate that these micellar systems weakly binds to the protein. Further, we believe that the binding is slightly enhanced by the presence of hydrophobic polycaprolactone moiety, which interacts with the hydrophobic domains of proteins.^21^ This protects the misfolded protein chains (which form because of the thermal stress) from aggregation by acting as molecular shields to prevent collisions between the misfolded chains and also provides an environment for the proteins to fold back to their native state. Once the protein returns to its native state, the interaction between the polymeric system and the protein decreases,^48^ and the protein in its native state can be isolated with centrifugation. These studies clearly show that these micellar systems have great ability to not only protect proteins from stress, but can also be easily and safely removed when required. Additionally, the micelles are biocompatible, with cell viability greater than 75% at all concentrations, indicating that they can be safely used in various biopharmaceutical applications (Fig. S19†).

## Conclusions

In summary, we demonstrated the preparation of novel glycopolymeric zwitterionic micelles composed of poly-SPB and trehalose by RAFT polymerization. The micelles show remarkably high ability to stabilize proteins under different forms of stress, and the proteins retain almost all their native properties in the presence of the micelles. More importantly, owing to the micellar structure, proteins can be safely recovered from the micelles with almost the same activity as that of the native proteins. To the best of our knowledge, this is the first study demonstrating the preparation of a scaffold-like micellar system for the stabilization of proteins, which could possibly be used for the development of off-the-shelf protein-based products, where a protein or antibody solution can be stored or transported under any condition (eliminating the need for low-temperature storage) with isolation whenever needed. Additionally, such systems can be used for protein or antibody purification or processing and for the production of recombinant proteins. Further studies on increasing the efficiency and testing of various proteins, antibodies, and mRNA are underway.

## Author contributions

Conceptualization, R. R. and K. M.; methodology, R. R.; funding acquisition, R. R. and K. M.; investigation, R. R.; formal analysis, R. R.; writing—original draft, R. R.; writing—review & editing, R. R. and K. M.

## Conflicts of interest

There are no conflicts to declare.

## Supplementary Material

NA-005-D3NA00002H-s001
