# Supplementary material for: Design of self-assembled glycopolymeric zwitterionic micelles as removable protein stabilizing agents
Source: Nanoscale Adv. 2023 Feb 21;5(6):1767–75. doi: 10.1039/d3na00002h (PMC10012880; doi:10.1039/d3na00002h)
Supplement: NA-005-D3NA00002H-s001 [file NA-005-D3NA00002H-s001.pdf]

Electronic Supplementary Material (ESI) for Nanoscale Advances.
This journal is © The Royal Society of Chemistry 2023

Electronic Supplementary information

Design of self-assembled glycopolymeric zwitterionic micelles as removable protein stabilizing agents

R. Rajan, and K. Matsumura**

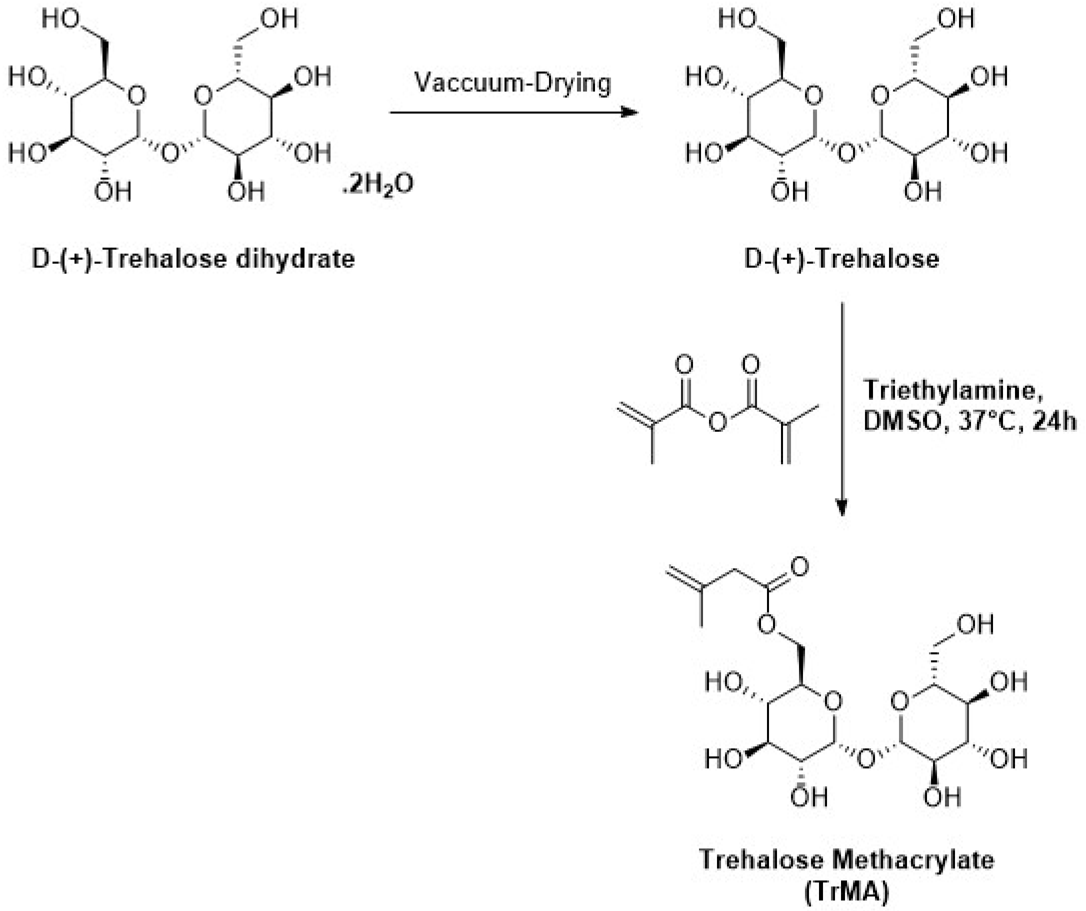

Scheme S1. Synthesis of trehalose methacrylate. Methacrylic anhydride was added to dried trehalose to substitute hydroxyl groups for a polymerizable methacrylate group.

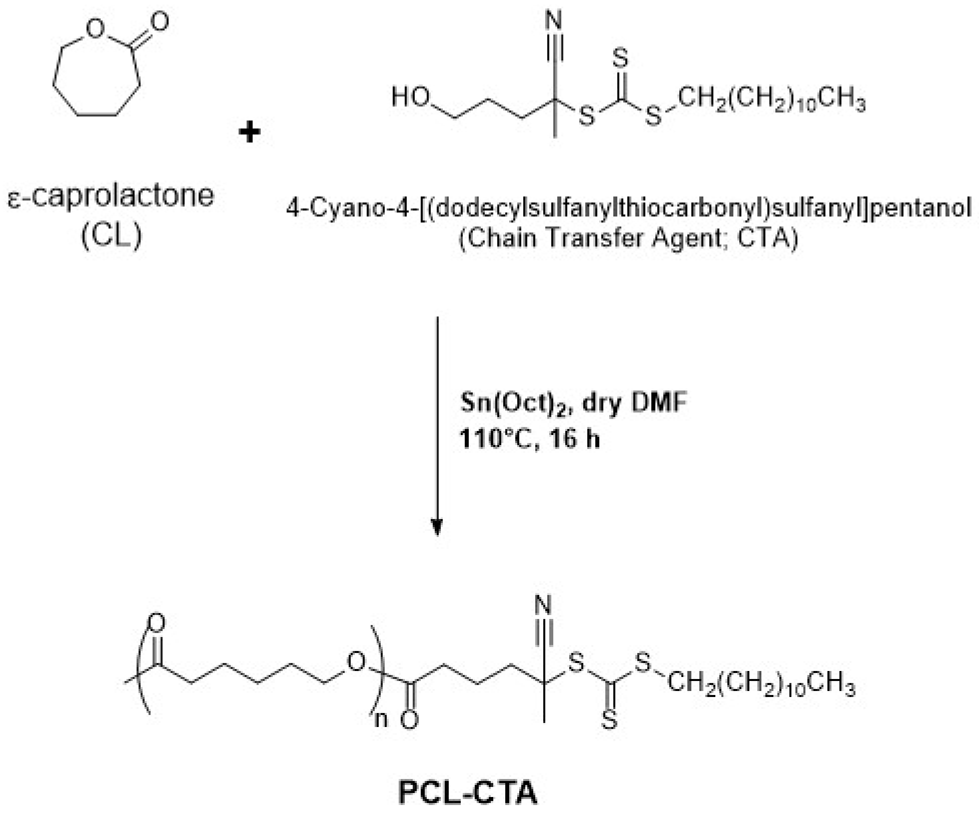

Scheme S2. Synthesis of poly-caprolactone (PCL). Ring opening polymerization of caprolactone using hydroxyl-terminated reversible addition–fragmentation chain-transfer (RAFT) agent in the presence of stannous(II) octoate.

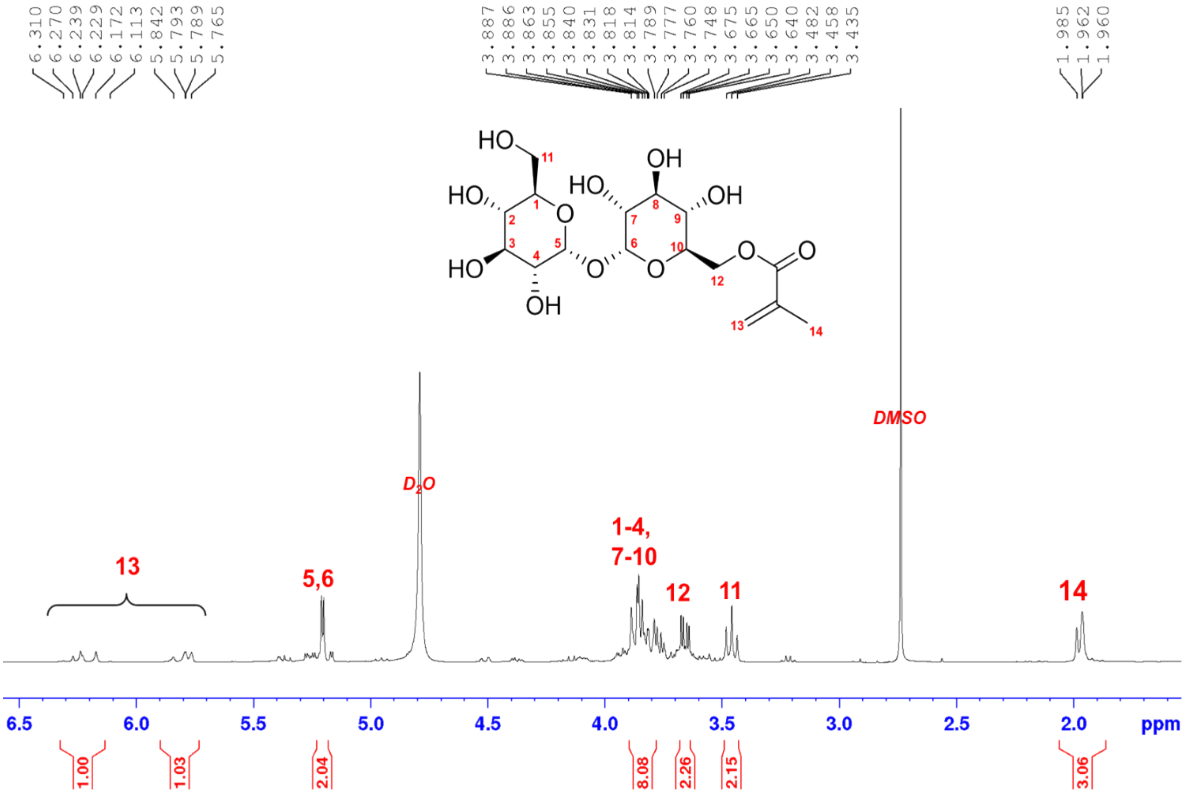

Fig. S1. ^{1}H NMR spectrum of TrMA in D_2O.

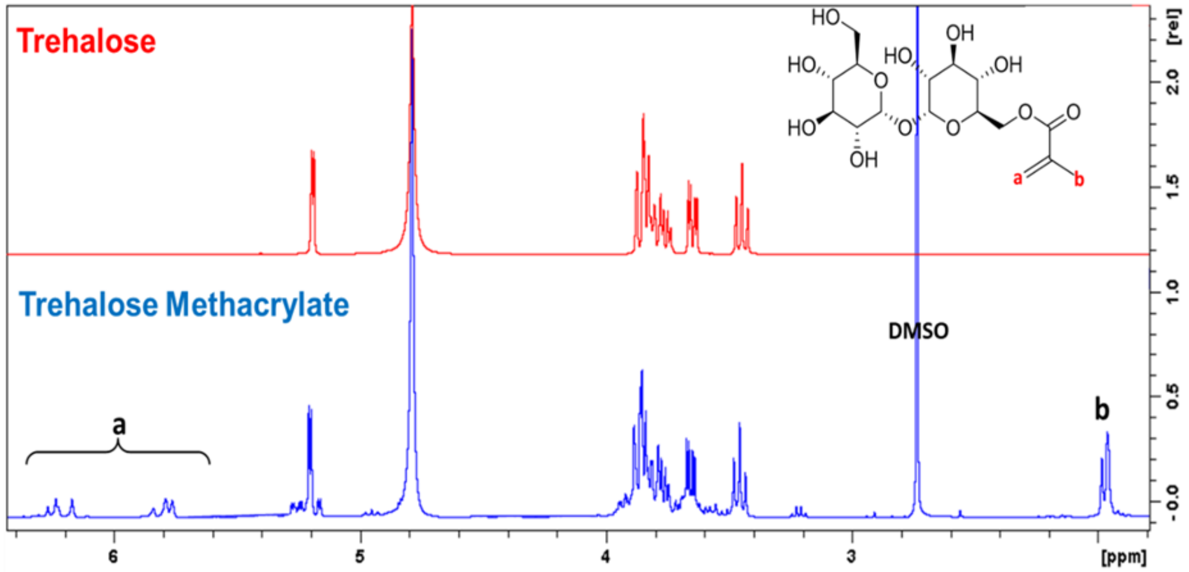

Fig. S2. Comparison of the ^{1}H NMR spectra (in D_2O) of trehalose and TrMA.

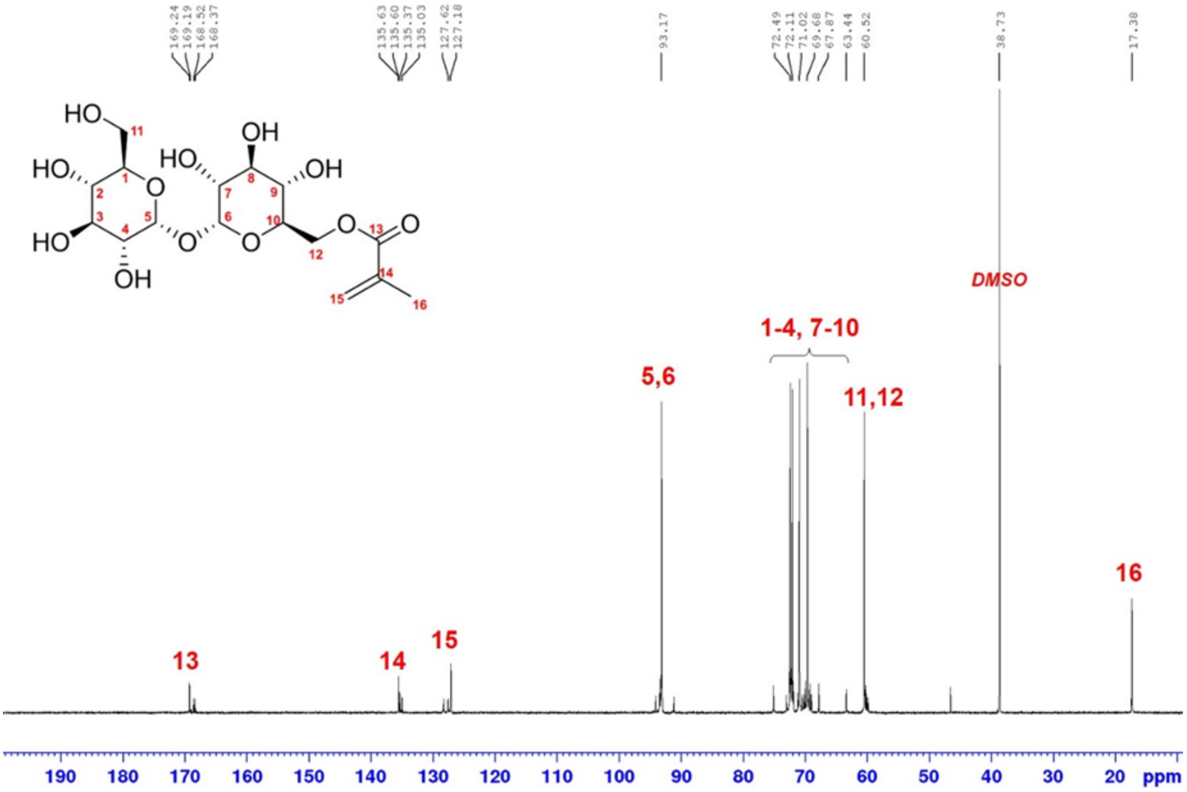

Fig. S3. ^{13}C NMR spectrum of TrMA in D_2O.

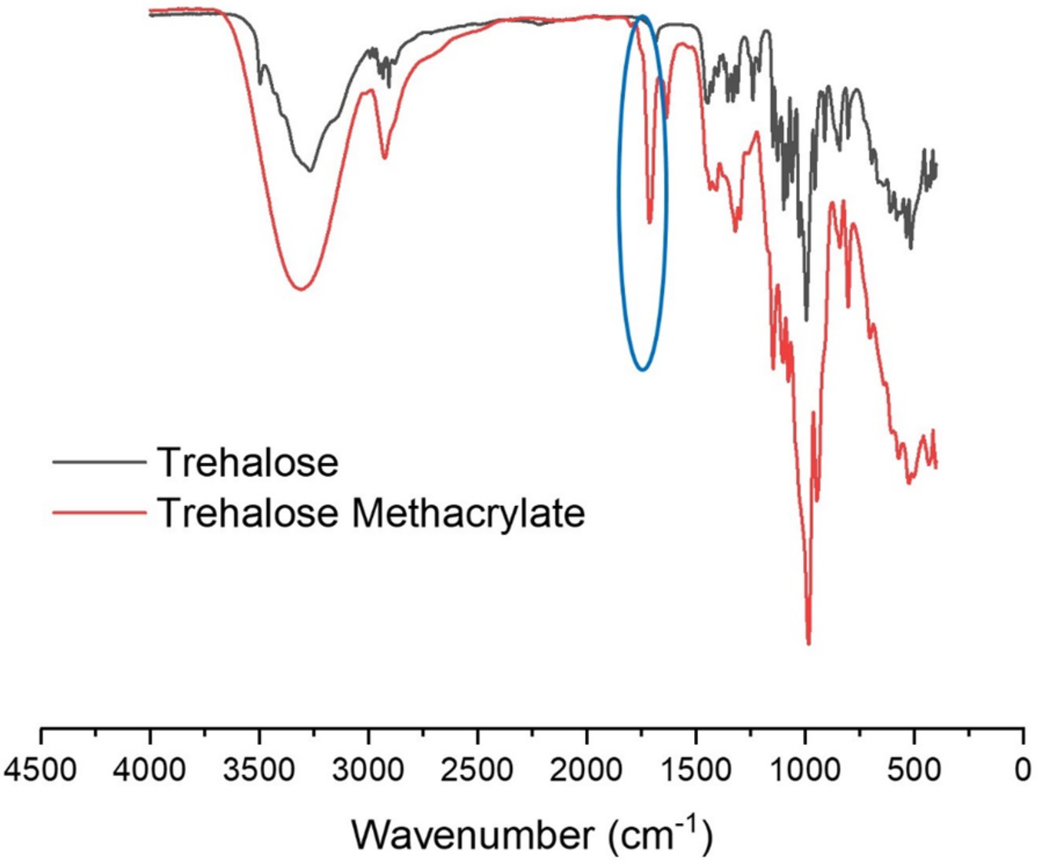

Fig. S4. Comparison of the FTIR spectra of trehalose and TrMA.

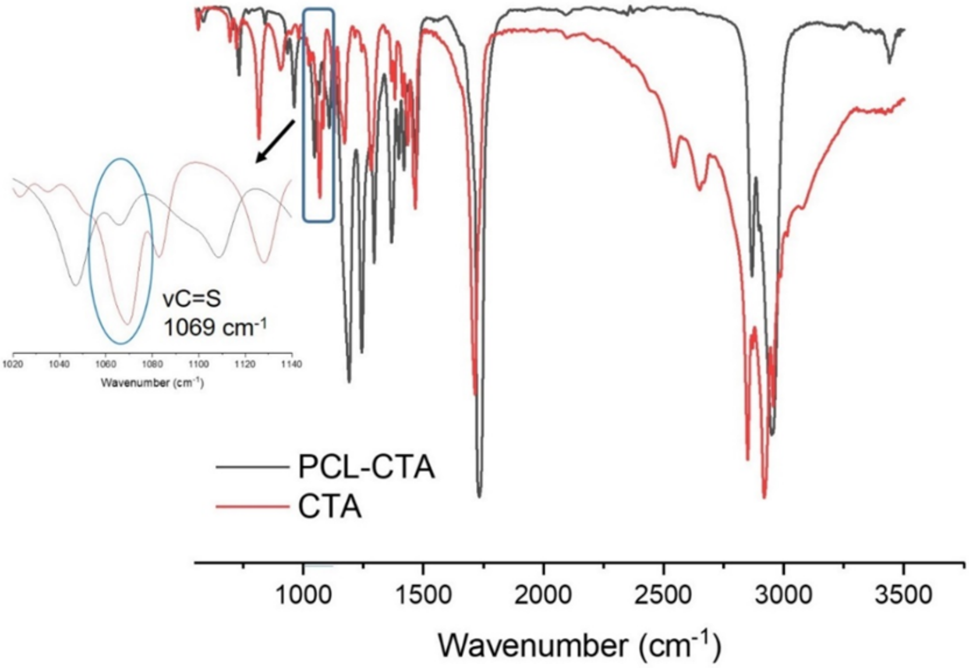

Fig. S5. Comparison of the FTIR spectra of PCL-CTA and CTA.

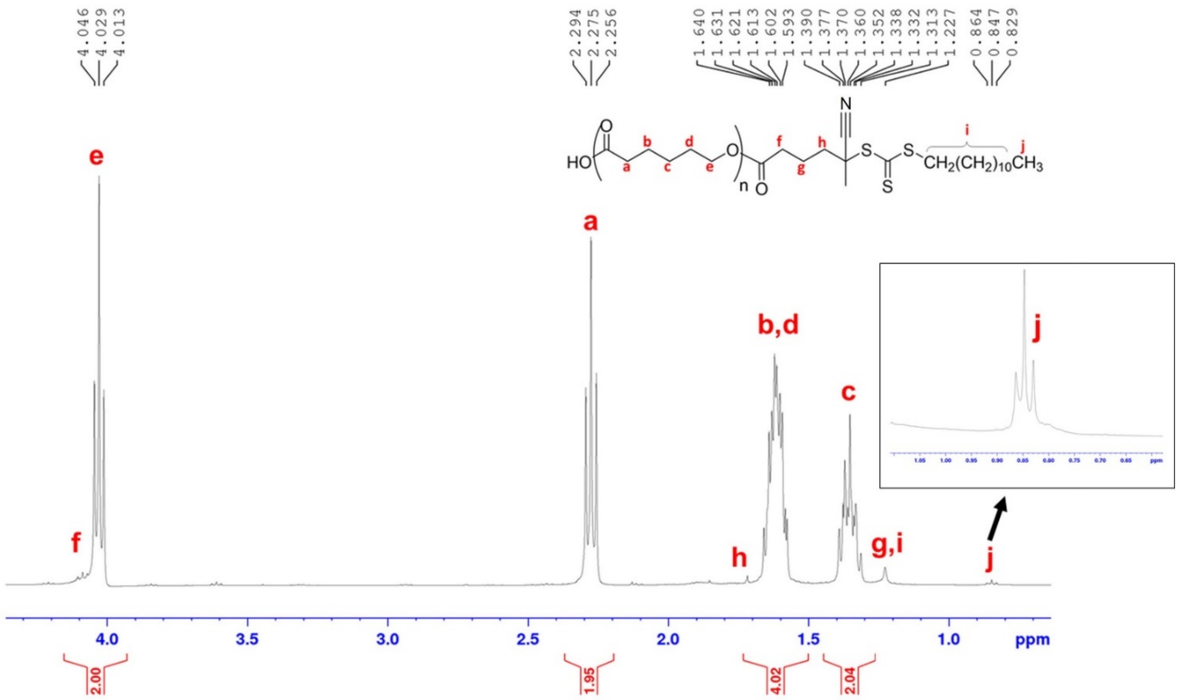

Fig. S6. ^{1}H NMR of PCL-CTA in $CDCl_3$.

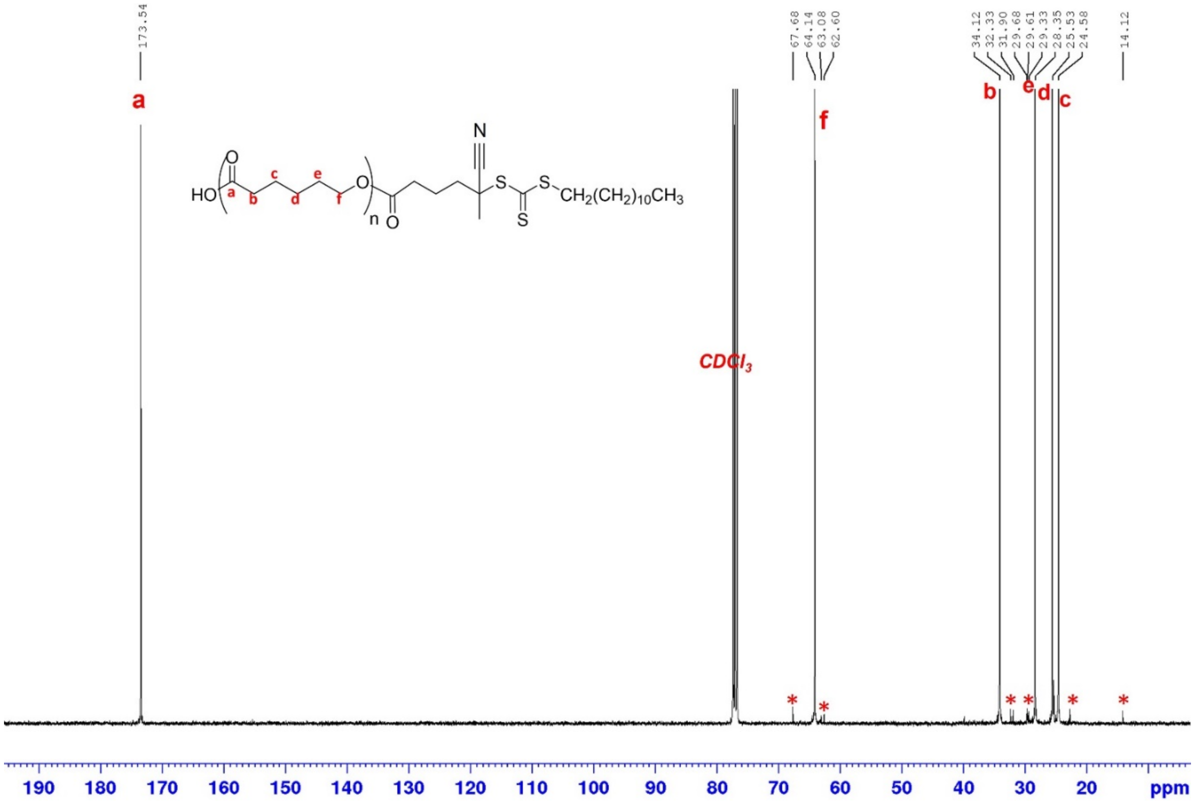

Fig. S7. ^{13}C NMR spectrum of PCL-CTA in $CDCl_3$ (* indicates RAFT agent peaks).

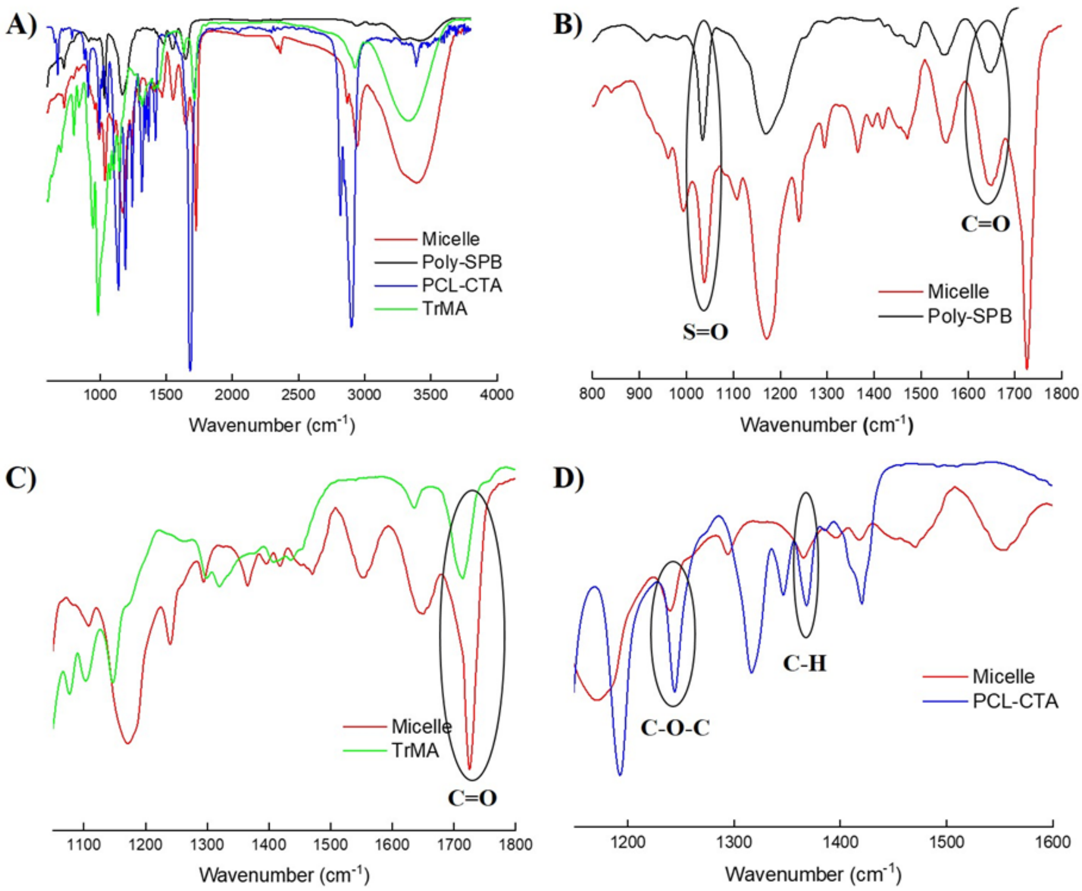

Fig. S8. Investigating the presence of all three components in the micelles. FTIR spectra of micelle and A) its corresponding components, B) poly-SPB, C) trehalose methacrylate, and D) PCL-CTA.

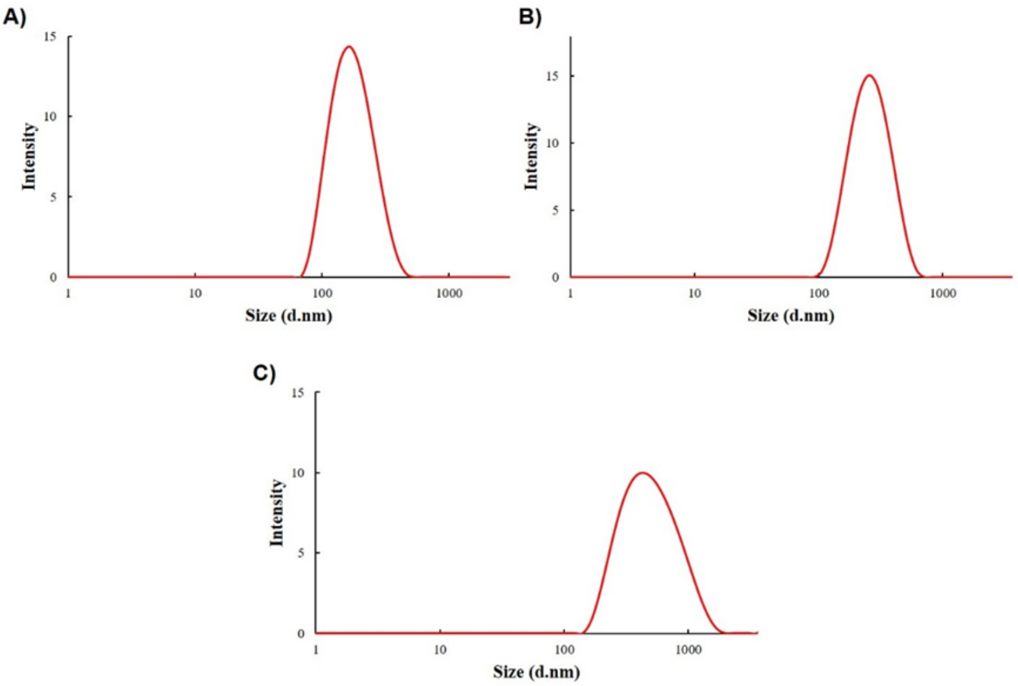

Fig. S9. Size of micelles. Size distribution by number obtained for the micelles by dynamic light scattering (DLS) for A) M1, B) M2, and C) M3.

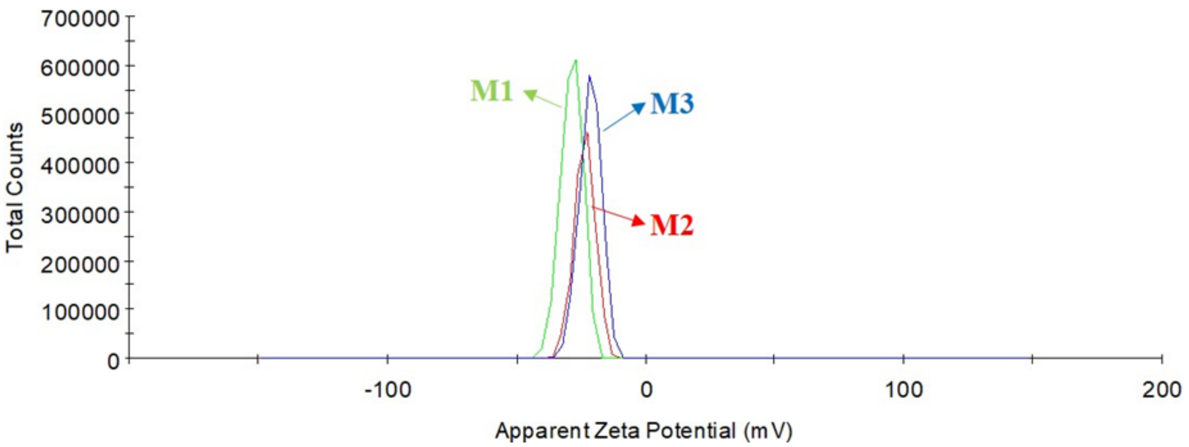

Fig. S10. Surface charge of micelles. Zeta potential distributions of the micelles were observed at 4 mg/mL in deionized water.

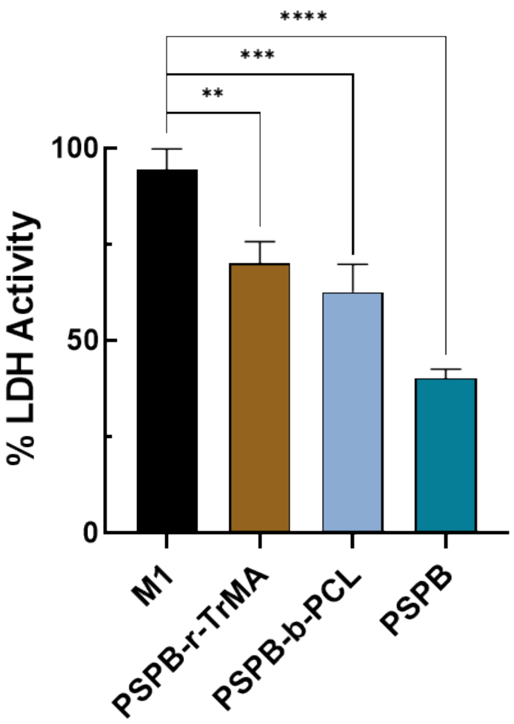

Fig. S11. Residual enzymatic activity. Enzymatic activity of LDH after incubation in presence of different additives (2 mg/mL) after incubation at 37 °C for 1 h.

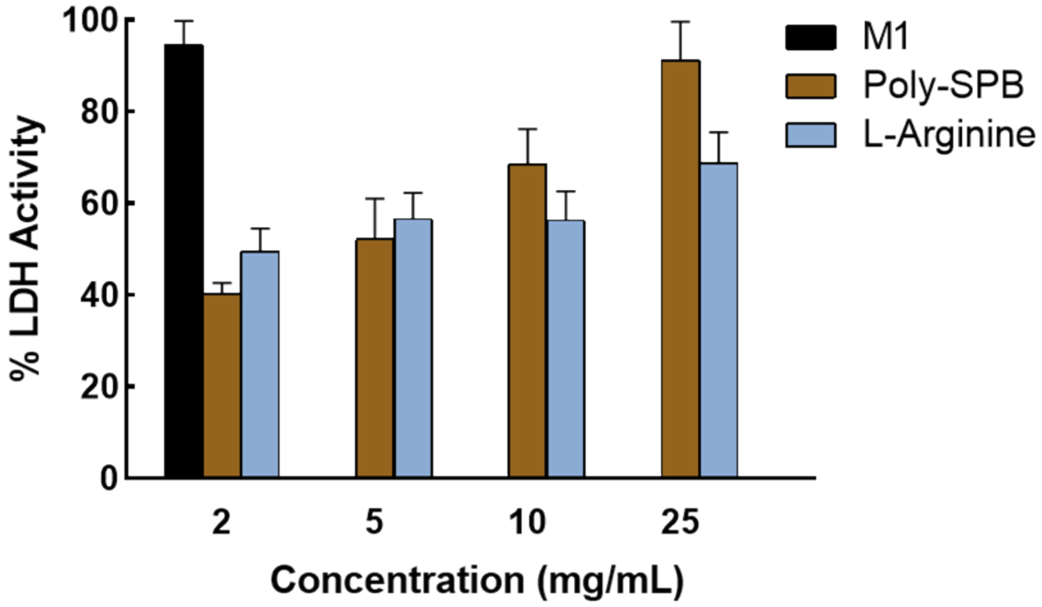

Fig. S12. Residual enzymatic activity. Enzymatic activity of LDH after incubation in the presence of different additives at different concentrations after incubation at 37 °C for 1 h.

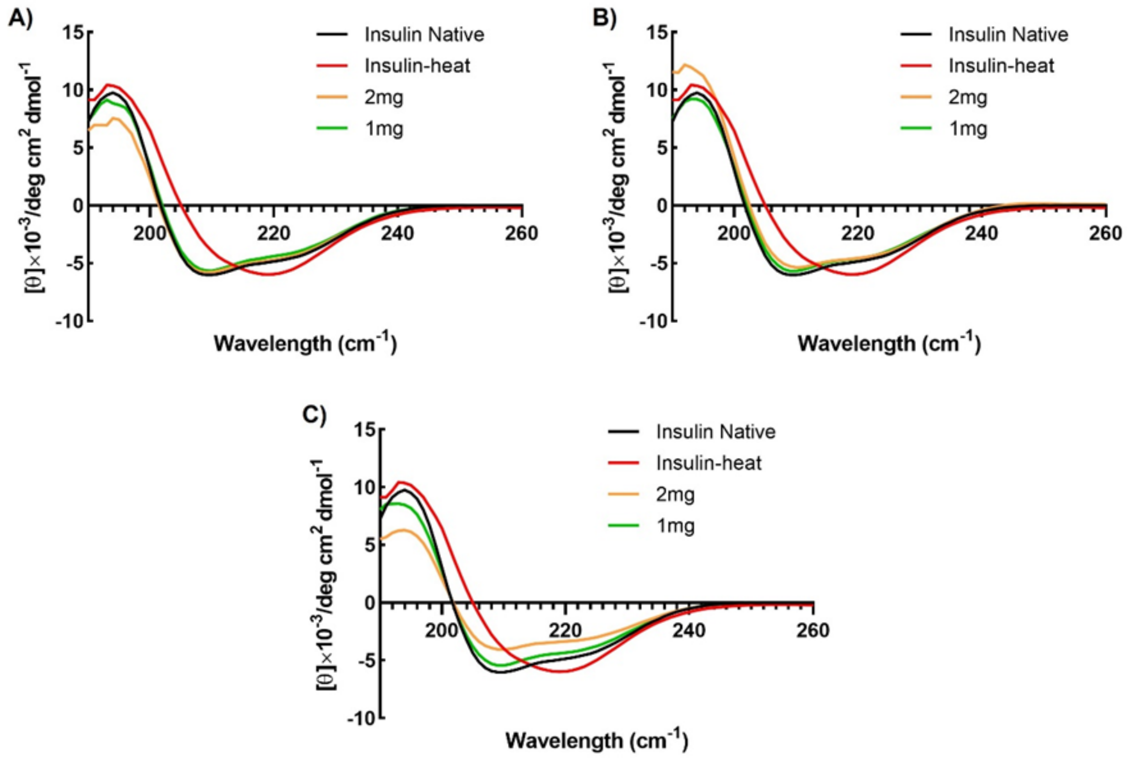

Fig. S13. Secondary structure of insulin. Representative far-UV CD spectra of human insulin after incubation at 45 °C for 72 h in the presence of A) M1, B) M2, and C) M3.

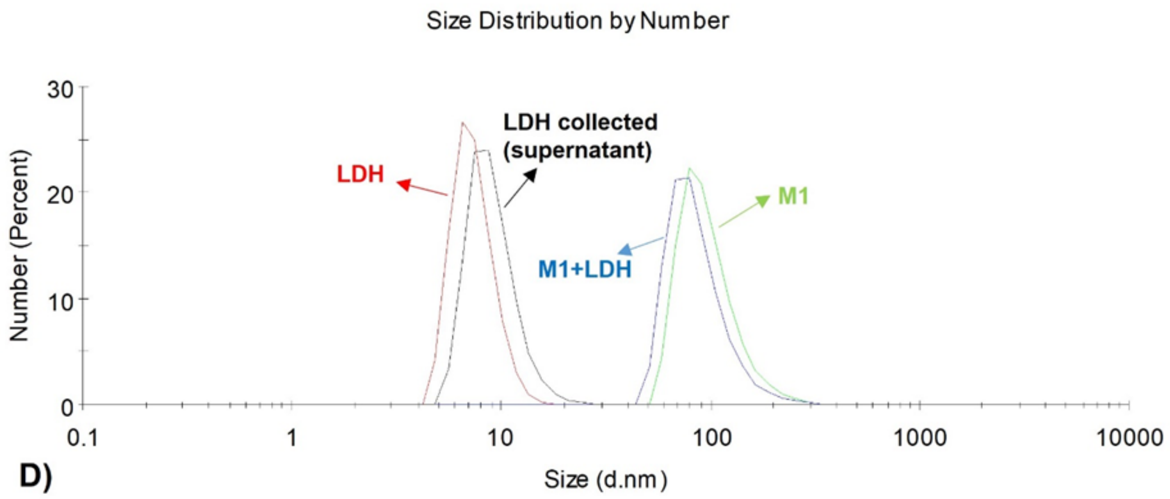

Fig. S14. Removal of micelles from protein. DLS analysis of micelles before and after centrifugation.

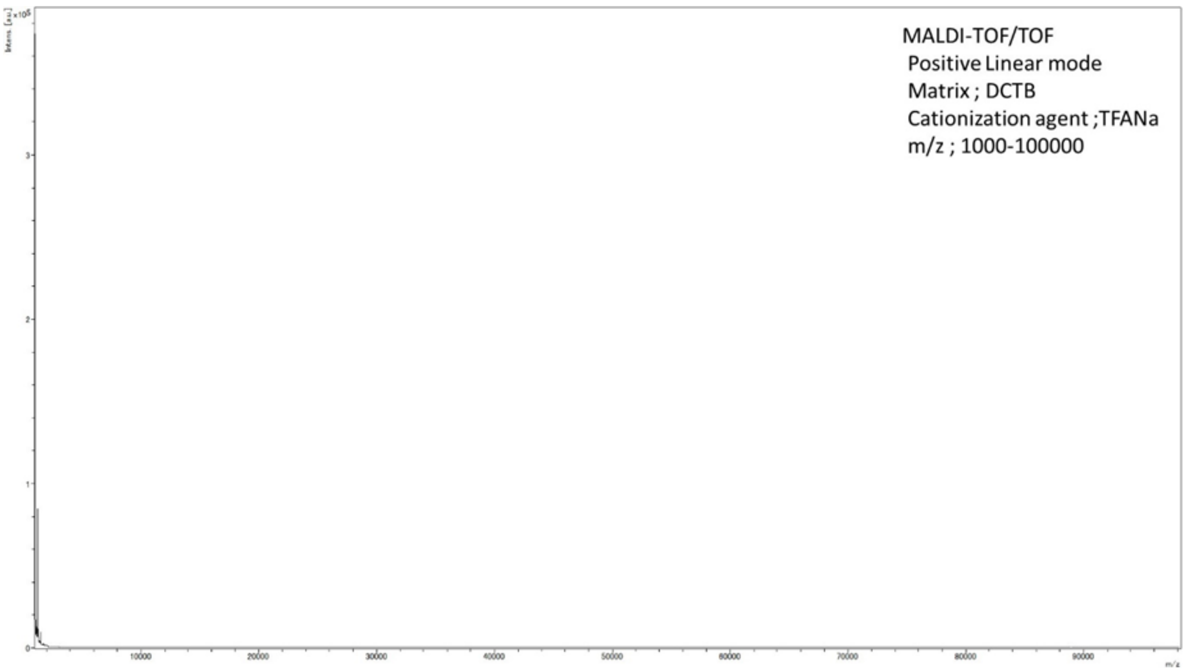

Fig. S15. Removal of micelles from protein**.** MALDI-TOF analysis of the supernatant recovered after centrifugation of micelles.

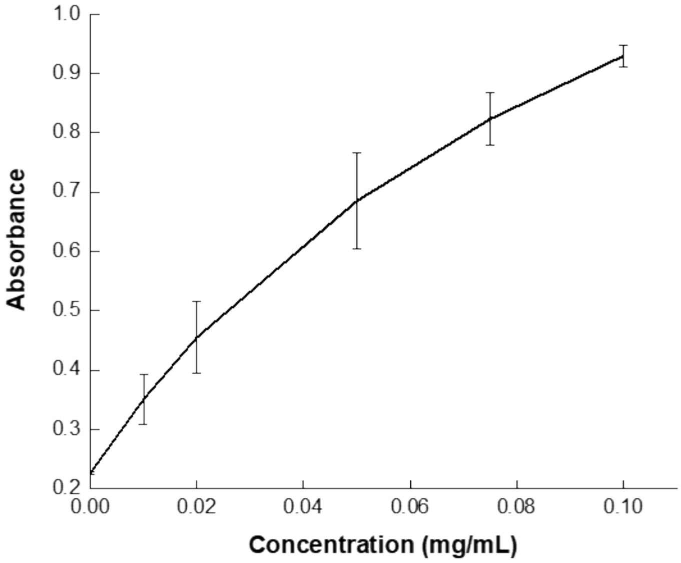

Fig. S16. LDH calibration curve. Bradford assay standard curve of LDH concentration versus absorbance (595 nm).

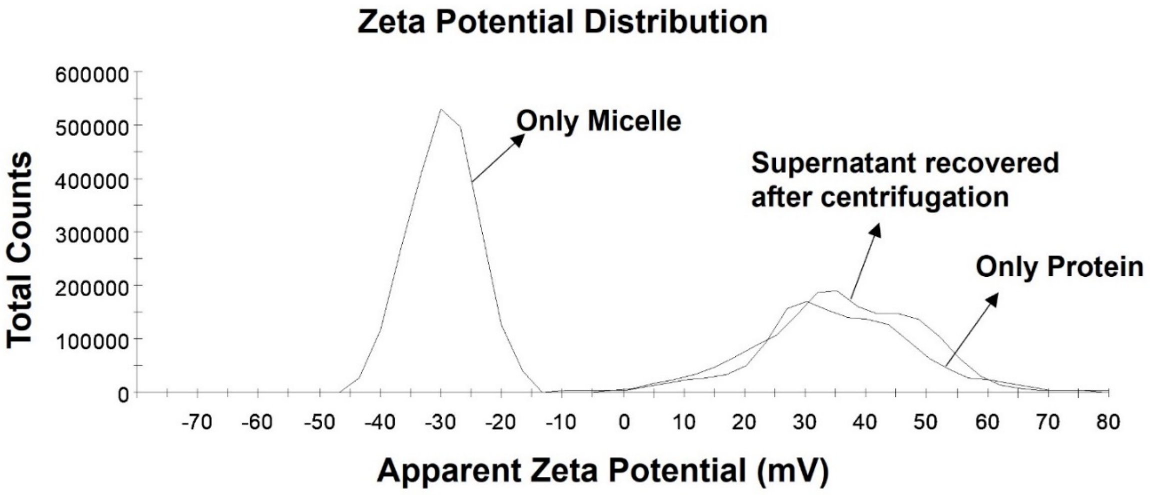

Fig. S17. Removal of micelles from protein. Zeta potential analysis of micelles before and after centrifugation.

Table S1. Summary of the recovery rates of LDH after ultracentrifugation.

	Protein recovered (μg)	Recovery rate (%)
**M1**	65.66	91.55 ± 2.83
**M2**	64.31	92.75 ± 1.87
**M3**	63.66	94.87 ± 1.29

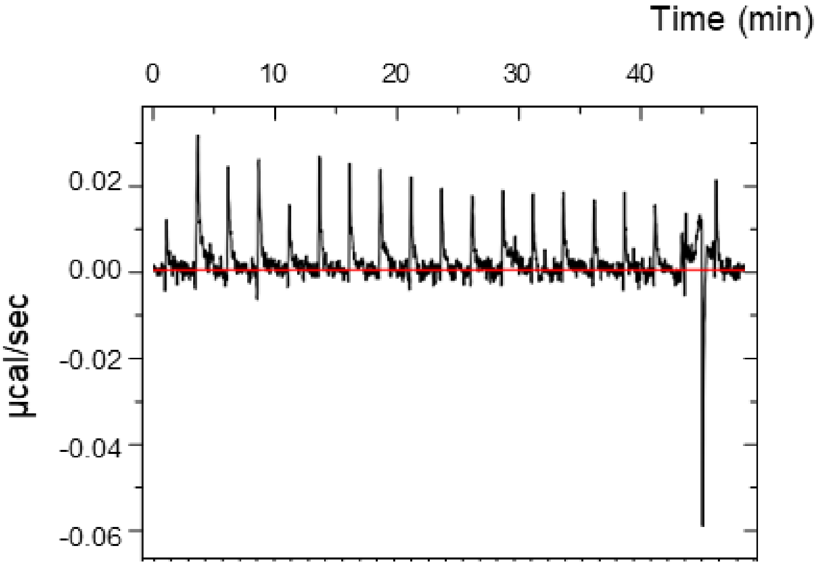

Fig. S18. Isothermal titration calorimetry results of the interaction between poly-SPB and insulin at 25°C.

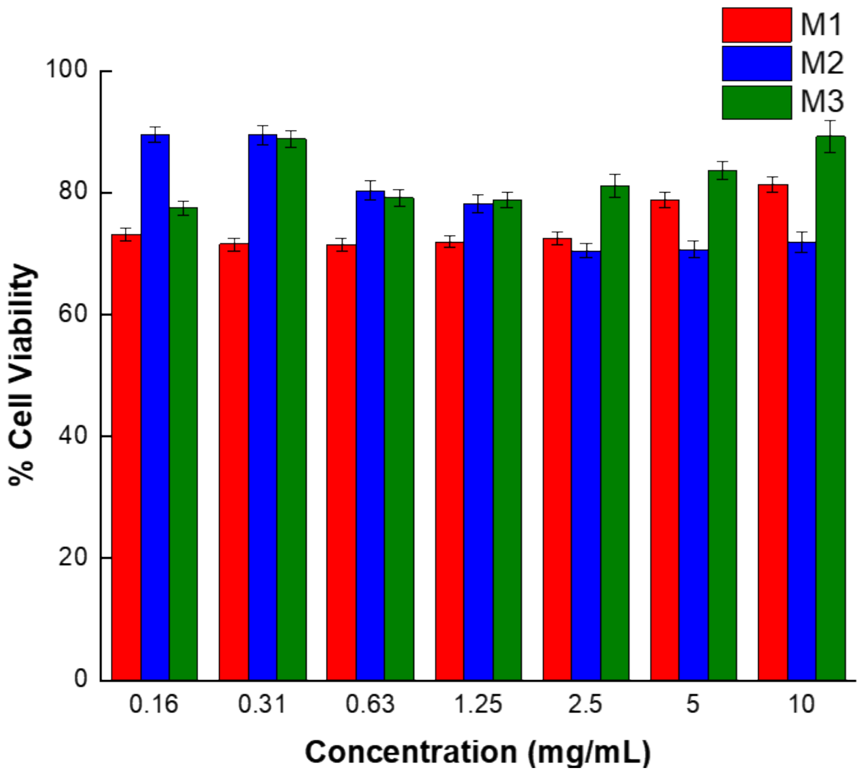

Fig. S19. Cytotoxicity of the micelles. The viabilities of L929 cells were tested after 24 h of treatment with different concentrations of micelles by MTT assay. Error bars indicate standard deviation of the mean.
